# Impaired Autophagy in Adult Bone Marrow CD34^+^ Cells of Patients with Aplastic Anemia: Possible Pathogenic Significance

**DOI:** 10.1371/journal.pone.0149586

**Published:** 2016-03-01

**Authors:** Jinbo Huang, Meili Ge, Shihong Lu, Jun Shi, Wei Yu, Xingxin Li, Min Wang, Jizhou Zhang, Sizhou Feng, Shuxu Dong, Xuelian Cheng, Yizhou Zheng

**Affiliations:** State Key Laboratory of Experimental Hematology, Institute of Hematology & Blood Diseases Hospital, Chinese Academy of Medical Science & Peking Union Medical College, Tianjin, P.R. China; B.C. Cancer Agency, CANADA

## Abstract

Aplastic anemia (AA) is a bone marrow failure syndrome that is caused largely by profound quantitative and qualitative defects of hematopoietic stem and progenitor cells. However, the mechanisms underlying these defects remain unclear. Under conditions of stress, autophagy acts as a protective mechanism for cells. We therefore postulated that autophagy in CD34^+^ hematopoietic progenitor cells (HPCs) from AA patients might be impaired and play a role in the pathogenesis of AA. To test this hypothesis, we tested autophagy in CD34^+^ cells from AA samples and healthy controls and investigated the effect of autophagy on the survival of adult human bone marrow CD34^+^ cells. We found that the level of autophagy in CD34^+^ cells from AA patients was significantly lower than in age/sex-matched healthy controls, and lower in cases of severe AA than in those with non-severe AA. Autophagy in CD34^+^ cells improved upon amelioration of AA but, compared to healthy controls, was still significantly reduced even in AA patients who had achieved a complete, long-term response. We also showed that although the basal autophagy in CD34^+^ cells was low, the autophagic response of CD34^+^ cells to “adversity” was rapid. Finally, impaired autophagy resulted in reduced differentiation and proliferation of CD34^+^ cells and sensitized them to death and apoptosis. Thus, our results confirm that autophagy in CD34^+^ cells from AA patients is impaired, that autophagy is required for the survival of CD34^+^ cells, and that impaired autophagy in CD34^+^ HPCs may play an important role in the pathogenesis of AA.

## Introduction

Acquired aplastic anemia (AA) is a bone marrow failure syndrome characterized by pancytopenia in the peripheral blood and bone marrow hypoplasia. Profound reduction in hematopoietic stem and progenitor cells has been a consistent finding in AA **[1–4]**. Additionally, at the time of clinical presentation, the number of long-term culture-initiating cells (LTC-ICs) is usually <10% of normal, and the number of stem cells has been estimated to be <1% of normal **[5]**. This damage to hematopoietic stem and progenitor cells may be due to a direct bone marrow insult or immune-mediated destruction **[6, 7]**. Profound qualitative defects to hematopoietic stem and progenitor cells are also a feature of most AA patients **[8–11]**. For example, hematopoietic progenitor cells (HPCs) from AA patients demonstrate decreased sensitivity to trophic signals **[12],** and a higher frequency of apoptosis than normal HPCs in the presence of inhibitory cytokines such as interferon-gamma (IFN-γ) **[13,14]**. They also have shorter telomeres measured by various methods **[15–17]** and reduced colony-forming cell (CFC) or LTC-IC activity of their CD34^+^ cells even in the presence of a high level of hematopoietic growth factors **[18]**. Despite these findings, the mechanisms underlying the defects in hematopoietic stem and progenitor cells from AA patients have not yet been elucidated.

Autophagy is a conserved proteolytic mechanism that acts as a protective mechanism under conditions of stress, and it maintains cellular integrity by regenerating metabolic precursors and clearing subcellular debris **[19–21]** while contributing to basal cellular and tissue homeostasis. Autophagy is involved in cell development, starvation adaptation, intracellular quality control, tumor suppression, aging, innate immunity and other processes **[22, 23]**. Abnormal autophagy has been demonstrated to be a direct cause of cell death **[23–25]** and has been implicated in infectious disease **[26, 27]**, cancer **[28]**, cardiovascular disease **[29]**, neurodegenerative disease **[30, 31]**, and metabolic and autoimmune disease **[32]**. Recent work demonstrated that autophagy is active in murine CD34^+^Flt3^−^ cells and adult CD34^+^CD133^+^ cells **[33, 34]**. Furthermore, autophagy in murine hematopoietic stem and progenitor cells is robustly induced after *ex vivo* cytokine withdrawal and *in vivo* calorie restriction **[35]**. Autophagy is also required for self-renewal and differentiation of CD34^+^CD133^+^ cells, and it serves as an adaptive stress response mechanism in hematopoietic stem and progenitor cells **[34, 36]**. However, there have been very few studies investigating autophagy in adult human bone marrow HPCs.

HPCs from AA patients are quantitatively and qualitatively defective, and they die more readily under stress. Thus, because autophagy can serve as a protective stress response pathway, we postulated that autophagy might be defective in CD34^+^ HPCs from AA patients.

## Methods

### Patients and controls

A total of 101 patients with acquired AA (38 females; 63 males; median age, 26.5 years; age range, 18 to 45 years) were included in the study between June 2012 and November 2015 at a single institution (Institute of Hematology & Blood Diseases Hospital, Chinese Academy of Medical Science & Peking Union Medical College). The cohort included 20 patients with *de novo* severe AA (SAA), 23 with *de novo* non-severe AA (NSAA) and 58 cases who had received prior treatment. The diagnosis and classification of AA was established according to the criteria of Camitta *et al* **[37, 38].** The control cohort included 38 age/sex-matched healthy volunteers (15 females; 23 males; age range, 19–45 years), and all patients and controls had given written informed consent for participation in accordance with the Helsinki Declaration. The study was approved by the Ethics Committees of the Institute of Hematology, Chinese Academy of Medical Sciences (CAMS) and Peking Union Medical College (PUMC) (Ethics number: KT2014005-EC-1).

### Cell isolation

Bone marrow mononuclear cells were separated by density-gradient centrifugation, and the following antibodies were used for fluorescence activated cell-sorting (FACS) analyses: CD34-APC, CD11b-PE, CD3-FITC and the appropriate isotypic controls (all from BD Biosciences, San Jose, CA). CD34^+^, CD3^+^ and CD11b^+^ were used as markers of HPCs, T lymphocytes and neutrophils, respectively. Cell sorting was performed on a 4-laser, 10-detector FACS Aria-III (BD Bioscience, San Jose, CA). For part of the healthy control bone marrow samples, CD34^+^ cells were freshly purified using a CD34/MACS isolation kit according to the manufacturer’s instructions (Miltenyi Biotec, Bergisch Gladbach, Germany). The cell fraction showing a CD34^+^ cell purity of 95%±5% was used for the subsequent experiments.

### NanoPro technology

At least 2,000 cells (samples that included <2,000 CD34^+^ cells were discarded) were sorted by FACS directly into a 384-well plate and lyzed in 2 μL of a solution consisting of Bicine/CHAPS Lysis Buffer (ProteinSimple, Santa Clara, CA) plus DMSO Inhibitor Mix (ProteinSimple, Santa Clara, CA) and Aqueous Inhibitor Mix (ProteinSimple, Santa Clara, CA) at 4°C for 30 min. Then, 6 μL Premix G2 (pH 3–10) (ProteinSimple, Santa Clara, CA) plus pI Standard Ladder 3 (ProteinSimple, Santa Clara, CA) were mixed with the lysate. Anti-LC3B primary antibody produced in rabbit (Sigma-Aldrich, Saint Louis, USA) was diluted 1:50 into antibody dilution buffer, while GAPDH (Cell Signaling Technology, Inc. MA, USA) was used as internal control and diluted 1:50 into antibody dilution buffer. Anti-human IgG-HRP secondary antibody (ProteinSimple, Santa Clara, CA) was diluted 1:100 into antibody dilution buffer, and mixed with luminol/peroxide at a 1:1 ratio. The NanoPro 1000 (ProteinSimple, Santa Clara, CA) was loaded and run according to the manufacturer’s specifications. Emitted light was quantified for 30, 60, 120, 240, 480 and 960 seconds. Compass software 2.5.11 (ProteinSimple, Santa Clara, CA) was used to identify and quantify chemiluminescent peaks and visually optimize tracings.

### Confocal laser scanning microscopy

Cytospins were made directly from FACS-sorted cells on non-coated slides. Cells were fixed in ice-cold 100% methanol for 15 min at -20°C, rinsed three times in phosphate buffered saline (PBS), and then blocked in Blocking Buffer (1XPBS/5% normal serum/0.3% Triton™ X-100) for 60 min. Indirect immunostaining was performed at 4°C overnight using the polyclonal anti-LC3 primary antibody (1:200, Cell Signaling Technology, Inc. MA, USA) and rinsed three times in PBS. Then specimens were incubated in anti-rabbit IgG-Cy3 secondary antibody (1:200, Sigma-Aldrich, Saint Louis, USA) for 2 h at room temperature in the dark. Slides were then stained with DAPI to label nuclei. The anti-fading fluorescent mounting medium (Vectorshield, Vector Laboratories, Inc. Burlingame, USA) was added, and the cells were covered with coverslips. The slides were then imaged by confocal laser scanning microscopy (PerkinElmer UltraVIEW Vox, Cambridge, UK). Volocity software 4.0 was used to identify and quantify fluorescence intensity.

### Monodansylcadaverine (MDC) staining

Cell-based MDC (Cayman Chemical, Ann Arbor, USA) was diluted 1:1,000 in the Cell-based Assay Buffer (Cayman Chemical, Ann Arbor, USA), and sorted cells were incubated with the MDC staining solution for 10 min at 37°C. Cells were then rinsed in Cell-based Assay Buffer, resuspended in 20 μL Cell-based Assay Buffer, dropped on non-coated slides, and covered with coverslips. The slides were analyzed immediately by confocal laser scanning microscopy (PerkinElmer UltraVIEW Vox, Cambridge, UK). Volocity software 4.0 was used to identify and quantify fluorescence intensity.

### Transmission electron microscopy (TEM)

Cells were resuspended in 0.2 mL 100% serum and fixed in 1.0 mL 2.5% phosphate-buffered glutaraldehyde at 4°C for 24 h. Cell blocks were then fixed in a mixture of 3% glutaraldehyde, 1.5% paraformaldehyde and 0.1 M sodium cacodylate buffer (pH 7.2) at 4°C for 4 h, followed by fixing in a mixture of 1% osmic acid and 1.5% ferrocyanatum Kalium at 4°C for 1.5 h. After dehydration in a graded series of ethanol and acetone, the cell block samples were embedded in TAAB epoxy resin. Ultrathin sections (70–75 nm) were collected on copper grids and stained with uranyl acetate and lead citrate. The cells were analyzed using an electron microscope (Oberkochen, Germany); 20 cells were photographed per sample. Quantification was performed by consecutively scoring 10 cells in these ultrastructural preparations from each cell population.

### Cell culture

CD34^+^ cells were grown in IMDM GlutaMAX™ medium (Gibco, Grand Island, USA) supplemented with 10% fetal bovine serum (FBS, Gibco, Grand Island, USA), 50 ng/mL SCF (PeproTech, Rocky Hill, USA), 50 ng/mL FL (PeproTech, Rocky Hill, USA), 20 ng/mL IL-3 (PeproTech, Rocky Hill, USA) and 20 ng/mL TPO (PeproTech, Rocky Hill, USA) at 37°C in a humidified atmosphere that included 5% CO_2_.

### Colony assays

Sorted CD34^+^ cells were seeded at a density of 400 cells/well in 24-well plates containing methylcellulose medium (methocult^TM^ H4230, Stem Cell Technologies, Inc. Vancouver, Canada) supplemented with 50 ng/mL SCF, 50 ng/mL FL, 20 ng/mL IL-3 and 20 ng/mL TPO. These cultures were incubated at 37°C in a humidified atmosphere that included 5% CO_2_. Colonies of >50 cells were scored after 10 d of culture, and experiments were performed in triplicate.

### Apoptosis detection by AnnexinV-FITC/PI assay

Sorted CD34^+^ cells were seeded in 12-well plate and treated with 3-Methyladenine (3-MA, Sigma-Aldrich, Saint Louis, USA) or chloroquine (CQ, Sigma-Aldrich, Saint Louis, USA) for 24 h. The level of apoptosis was then determined by an Annexin V-FITC Apoptosis Detection Kit (BD Biosciences, San Jose, CA) according to the manufacturer’s instructions. Data acquisition was performed using a LSR II flow cytometer (BD Biosciences, San Jose, CA) and analyzed by TreeStar FlowJo software (Version7.6, Ashland, OR).

### Trypan blue assay

Sorted CD34^+^ cells were seeded into 12-well plates, treated with 3-MA or CQ for 48 h before harvest, and resuspended in PBS. Resuspended cells were then mixed with 0.4% Trypan Blue solution (Sigma-Aldrich, Saint Louis, USA) for 3 min at room temperature. The number of stained cells was then quantified with a hemocytometer using bright field microscopy.

### Statistical analysis

Analysis of variance (ANOVA) was used for statistical analysis, and all statistical analyses were performed with SPSS 17 software. A *P* value < 0.05 was considered statistically significant.

## Results

### Impaired autophagy in CD34^+^ cells from AA patients

To examine the autophagic activity in CD34^+^ cells from AA patients, we assessed their levels of microtubule-associated protein 1A/1B-light chain 3 (MAP1LC3B), an autophagy-related marker using the NanoPro1000^TM^ system (ProteinSimple, Santa Clara, CA). The NanoPro1000^TM^ system makes use of an ultrasensitive capillary electrophoresis immuno-detection instrument that allows proteins to be detected at >100-times higher sensitivity than conventional Western blot analysis, thus allowing clear identification of low abundance proteins that would not be detectable by Western blot analysis. Conversion of LC3-I to LC3-II by the addition of phosphatidylethanolamine is essential for the formation of autophagosomes, so LC3-II can serve as a marker of these cellular structures **[39–41]**. With the addition of phosphatidylethanolamine, the isoelectric point (pI) of LC3-II is different from LC3-I, thus LC3-I and LC3-II can be detected by the NanoPro1000^TM^ system at the same time.

**Fig 1:**
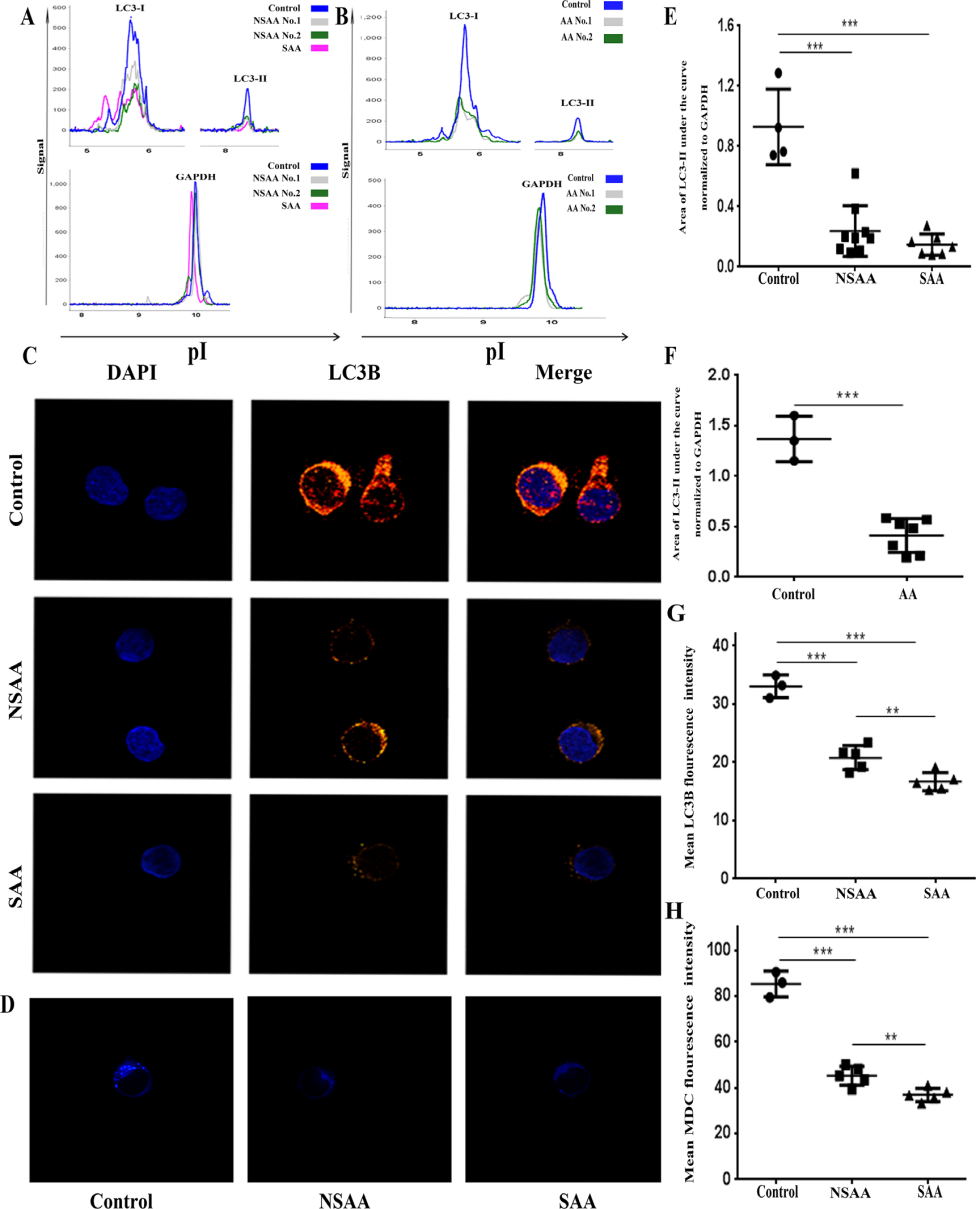
Impaired autophagy in CD34^+^ cells from AA patients. **(A) and (E):** NanoPro1000^TM^ system analysis. Samples were analyzed immediately after isolation without being first cultured. The peaks at pI 5.70, pI 8.36 and pI 9.95 represent LC3-I, LC3-II and GAPDH, respectively. The ratio of the peak area of LC3-II normalized to GAPDH from the same sample is presented, demonstrating that healthy controls had higher LC3-I/LC3-II than NSAA/SAA (SAA group, n = 7; NSAA group, n = 9; control group, n = 4). **(B) and (F):** NanoPro1000^TM^ system analysis. CD34^+^ cells were cultured in the presence of 20 μM CQ for 24 h prior to analysis. The addition of CQ accumulated LC3-II expression in all groups, though healthy controls had higher LC3-I/LC3-II than did AA (AA group, n = 7; control group, n = 3). **(C) and (G)**: Confocal microscopy (×1,000). Samples were analyzed immediately after isolation without being first cultured. The LC3B fluorescence density of each sample is shown. Healthy controls exhibited punctate LC3B (red), NSAA/SAA showed a weak cytosolic LC3B distribution, and the LC3B fluorescence density in the NSAA/SAA group was significantly lower than in the control group (SAA group, n = 5; NSAA group, n = 5; control group, n = 3). **(D) and (H):** Confocal microscopy (×1,000). Samples were analyzed immediately after isolation without being first cultured. The MDC fluorescence density of each sample is shown. Healthy controls exhibited more MDC-labeled particles (blue) and MDC fluorescence density than did the NSAA/SAA (SAA group, n = 5; NSAA group, n = 5; control group, n = 3). * *P*<0.05, ** *P*<0.01, *** *P*<0.001.

First, we tested LC3 in CD34^+^ cells from fresh samples. Results from this experiment showed that, compared with age/sex-matched healthy controls, the mean level of LC3-II in CD34^+^ cells from AA patients was significantly lower (*P<0*.*001*), for both SAA (0.15±0.07 vs 0.93±0.25, *P<0*.*001*) and NSAA (0.24±0.17 vs 0.93±0.25, *P<0*.*001*) samples. Interestingly, CD34^+^ cells from SAA patients had lower LC3-II levels than those from NSAA (0.15±0.07 vs 0.24±0.17), although this difference was not significant (*P* = 0.283) **(Fig 1A and 1E)**. We subsequently assayed LC3 in CD34^+^ cells that were cultured in the presence of 20 μM CQ for 24 h. CQ was used to help us explore changes in autophagic flux by inhibiting fusion of the autophagosome with the lysosome. Our results showed that the addition of CQ caused LC3-II expression to increase, but compared with healthy controls, the mean level of LC3-II in CD34^+^ cells from AA patients was still significantly lower (0.41±0.17 vs 1.37±0.22, *P<0*.*001*) **(Fig 1B and 1F)**. These results suggest autophagy is reduced in CD34^+^ cells from AA patients.

To further test the above result, we assessed autophagy in freshly sorted CD34^+^ cells by LC3B and MDC staining, two classic methods used to detect autophagy. The LC3-phosphatidylethanolamine pathway is involved in the formation of autophagosomes and autolysosomes, and LC3B can serve as a marker to evaluate the level of autophagy **[42]**. Here, CD34^+^ cells from healthy controls exhibited a significantly higher LC3B fluorescence intensity than that of AA cells, including cells from SAA (16.7±1.6 vs 33.04±1.94, *P<0*.*001*) and NSAA (20.8±2.1 vs 33.04±1.94, *P<0*.*001*) cases **(Fig 1C and 1G)**, confirming a reduced level of autophagosomes in CD34^+^ cells from AA patients. This result was further supported by staining with the fluorescent dye MDC, which interacts with lipids on autophagic vacuole membranes to enable autophagic vacuoles to be visualized **[43]**. Here, CD34^+^ cells from healthy controls exhibited more MDC-labeled particles and a higher mean MDC fluorescence intensity than those from AA patients (*P<0*.*001*) **(Fig 1D and 1H)**.

### Improved autophagy in CD34^+^ cells from AA patients with the amelioration of disease

**Fig 2:**
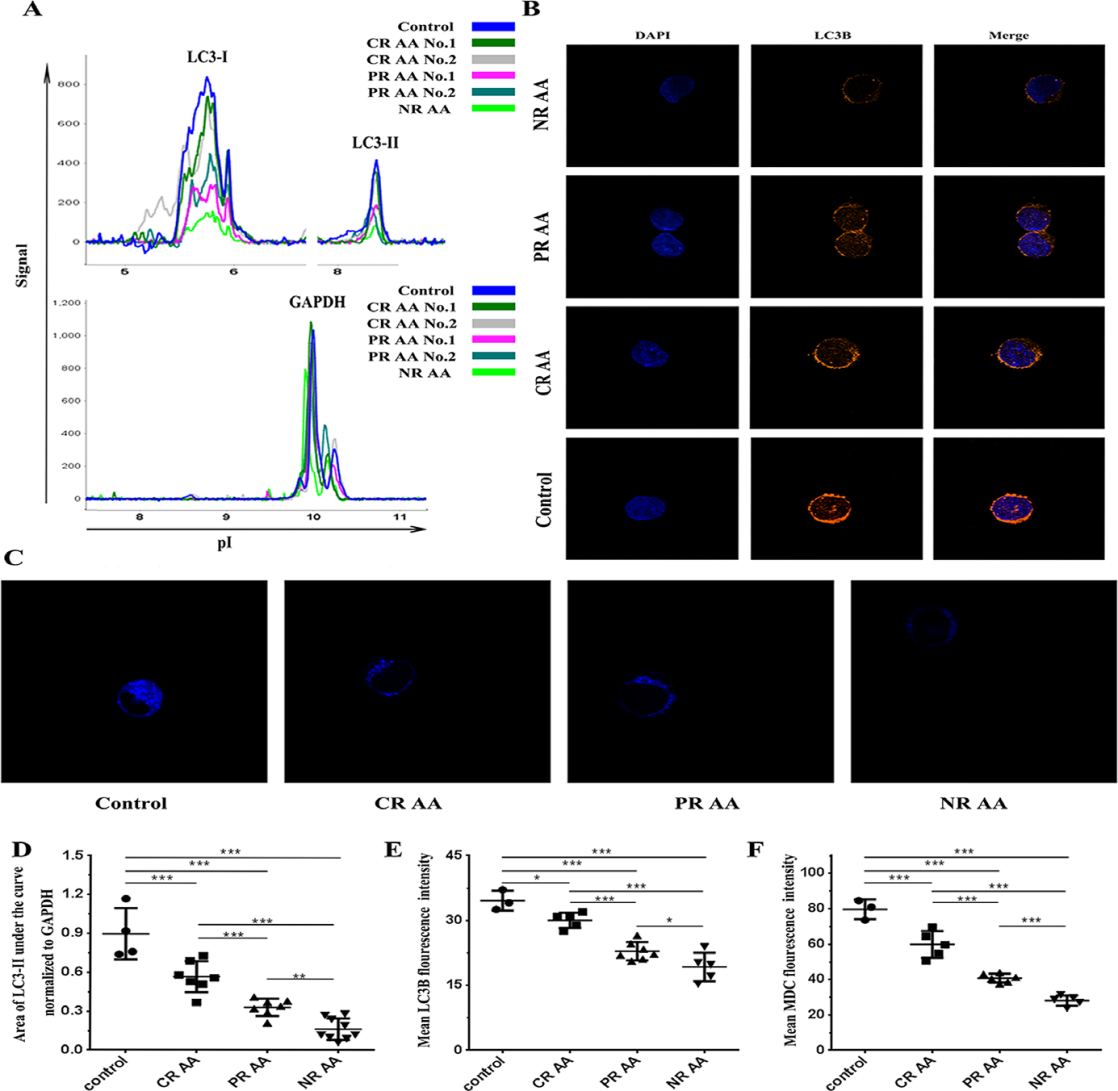
Improved autophagy in CD34^+^ cells from AA patients with the amelioration of disease. All freshly sorted CD34^+^ cells were analyzed immediately after isolation without culture. **(A) and (D)**: NanoPro1000^TM^ system. The LC3-I and LC3-II peak and the ratio of the LC3-II peak area normalized to GAPDH of each sample is presented (NR AA group, n = 9; PR AA group, n = 7; CR AA group, n = 7; control group, n = 4). With the amelioration of AA, the level of LC3-II increased but was still lower than in healthy controls. **(B) and (E)**: Confocal microscopy (×1,000). The LC3B fluorescence and mean LC3B fluorescence density of CD34^+^ cells is shown (NR AA group, n = 5; PR AA group, n = 7; CR AA group, n = 5; control group, n = 3). With the amelioration of AA, the mean LC3B fluorescence density increased, but was still lower than in healthy controls. **(C) and (F)**: Confocal microscopy (×1,000). The MDC fluorescence and mean MDC fluorescence density of CD34^+^ cells is shown (NR AA group, n = 5; PR AA group, n = 6; CR AA group, n = 7; control group, n = 3). With the amelioration of AA, the mean MDC fluorescence density increased, but was still lower than in healthy controls. * *P*<0.05, ** *P*<0.01, *** *P*<0.001.

Immunosuppressive treatment (IST) is most widely used for AA patients who are not eligible for allogeneic stem cell transplantation, and it leads to different responses, including no response (NR), partial response (PR) and complete response (CR). We assessed levels of LC3-II in freshly sorted CD34^+^ cells from different AA samples displaying all three responses. The evaluation of clinical response followed the 1998 Santa Margherita Ligure International Standards **[44]**. Our results revealed that the mean level of LC3-II in NR, PR and CR cases was 0.16±0.08, 0.33±0.07 and 0.57±0.12, respectively, and the difference between them was significant (PR vs NR, *P* = 0.007; CR vs PR, *P* = 0.001). The level of LC3-II was elevated with the amelioration of AA. However, compared with age/sex-matched healthy controls, it was still significantly decreased, even in long-term CR cases (0.57±0.12 vs 0.90±0.20, *P<0*.*001*) **(Fig 2A and 2D)**, suggesting that impaired autophagy in CD34^+^ cells from AA patients could partially recover with the amelioration of disease. This result was confirmed by LC3B and MDC staining. The mean LC3B fluorescence densities of NR, PR, CR AA and healthy controls were 19.3±3.3, 22.9±2.1, 30.0±1.8 and 34.6±2.3, respectively, with significant differences (PR vs NR, *P* = 0.021; CR vs PR, *P<0*.*001*; healthy control vs CR, *P* = 0.019) **(Fig 2B and 2E)**. The mean MDC fluorescence densities in NR, PR, and CR cases and healthy controls were 28.1±2.9, 40.7±2.5, 59.8±7.6 and 79.7±5.6, respectively, with differences that were also significant **(Fig 2C and 2F)**.

### Autophagic heterogeneity in CD34^+^ cells from AA patients

**Fig 3:**
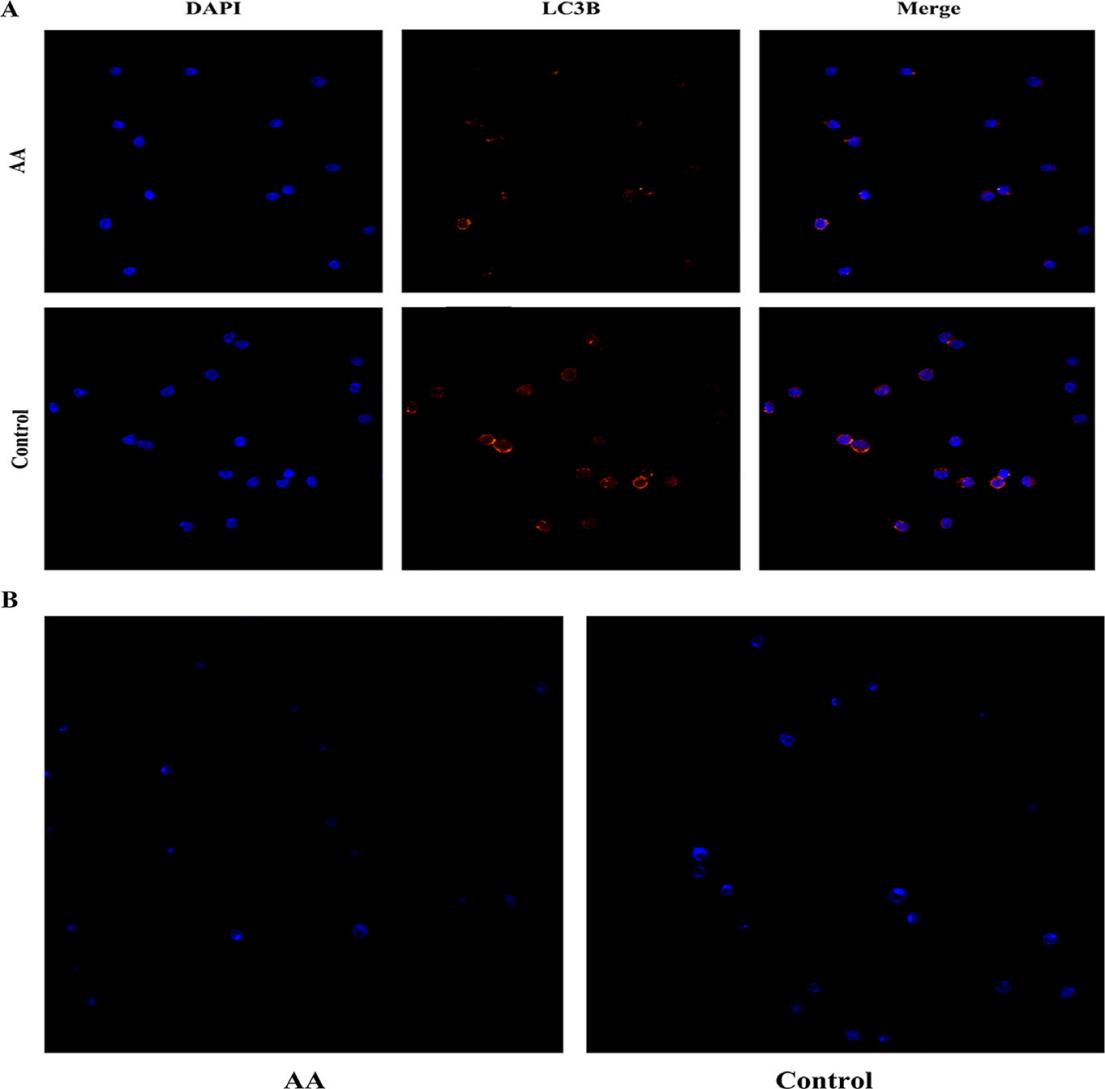
Autophagic heterogeneity in CD34^+^ cells from AA patients. **(A):** LC3B staining imaged with confocal microscopy (×200). A few residual CD34^+^ cells from AA patients showed a punctuate immunostaining pattern, just like CD34^+^ cells from healthy controls. **(B):** MDC staining by confocal microscopy (×200). A few residual CD34^+^ cells from AA patients contained MDC-labeled particles, just like CD34^+^ cells from healthy controls.

The mean level of autophagy in CD34^+^ cells from AA patients was significantly lower than that in healthy controls. However, we found that although most CD34^+^ cells from AA patients exhibited a weak LC3B immunostaining pattern, a few showed a punctate pattern similar to that seen in CD34^+^ cells from healthy controls **(Fig 3A)**. To show this variability in autophagy, we ranked the LC3B fluorescence density based on 500 healthy control CD34^+^ cells that came from 5 different samples. As shown in **Table 1**, in the healthy control group, the rate of CD34^+^ cells with a fluorescence density greater than 20 was 91.3%, while in the SAA and NSAA groups, the rates were 23.6% and 32.4%, respectively. This result indicated that there were a few residual CD34^+^ cells from AA patients with normal autophagic activity. Additionally, this finding was supported by MDC staining. We ranked the MDC fluorescence density based on the healthy control CD34^+^ cells and found that a few residual CD34^+^ cells from AA patients showed normal MDC-labeled particles and MDC fluorescence density **(Fig 3B and Table 1)**.

**Table 1:** Fluorescence density distribution of 250 CD34^+^ cells from 5 samples each from SAA and NSAA patients, and healthy controls.

Fluorescence density	SAA, n (%)	NSAA, n (%)	Control, n (%)
**LC3B**			
**≤20**	**191(76.4)**	**169(67.6)**	**22(8.7)**
**21–30**	**37(14.8)**	**41(16.4)**	**90(36.0)**
**31–40**	**19(7.6)**	**27(10.8)**	**83(33.3)**
**>40**	**3(1.2)**	**13(5.2)**	**55(22.0)**
**MDC**			
**≤60**	**215(86.0)**	**194(77.6)**	**25(10.0)**
**61–80**	**18(7.2)**	**25(10.0)**	**72(28.7)**
**81–100**	**13(5.2)**	**22(8.8)**	**108(43.3)**
**>100**	**4(1.6)**	**9(3.6)**	**45(18.0)**

### High autophagic activity in human bone marrow CD34^+^ cells

**Fig 4:**
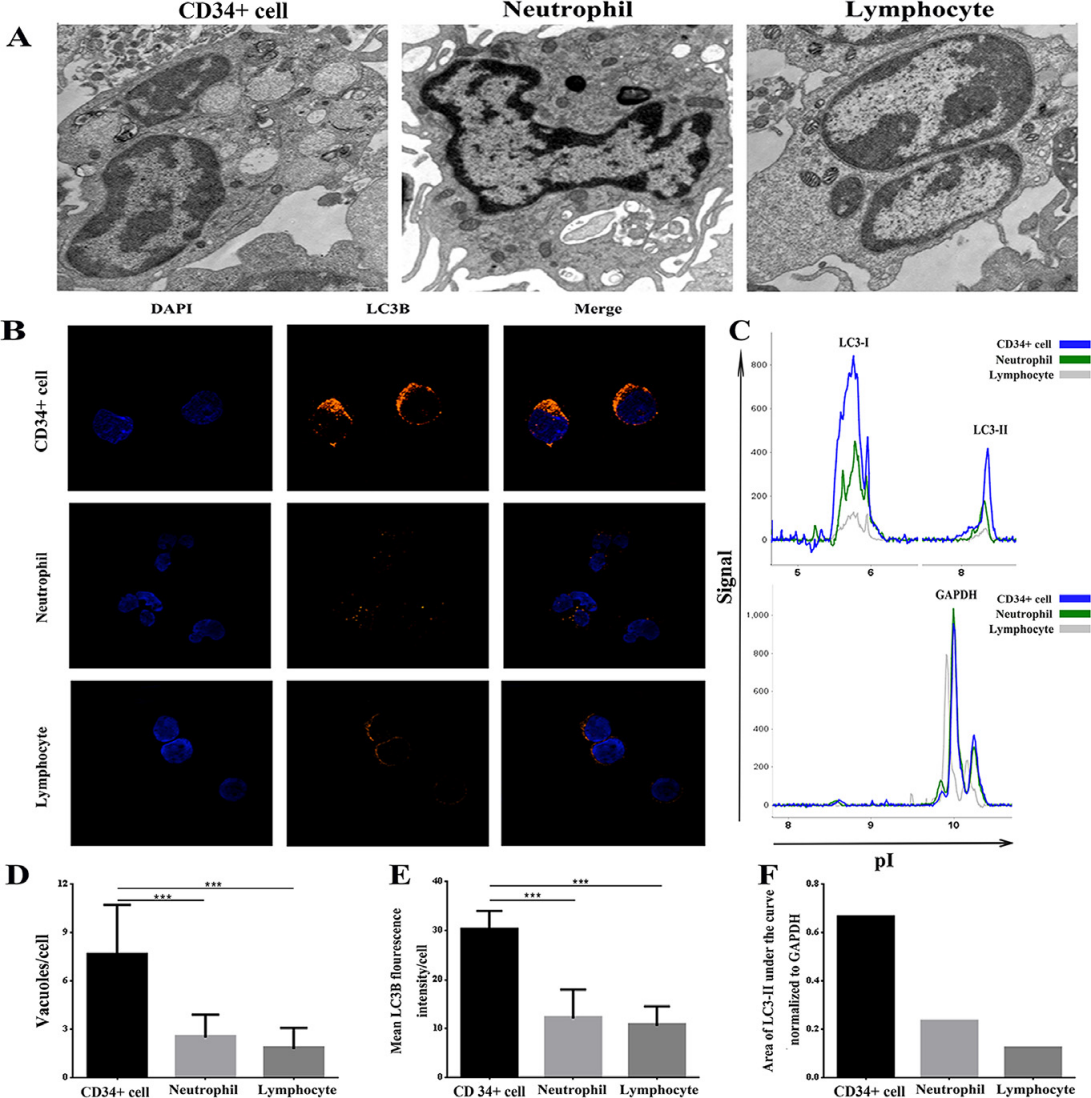
Freshly sorted normal adult human bone marrow CD34^+^ cells had higher autophagic activity than lymphocytes and neutrophils. **(A) and (D):** TEM (×10,000). Autophagosomes and the mean number of vacuoles (autophagosomes) in CD34^+^ cells, lymphocytes and neutrophils. CD34^+^ cells contain more vacuoles (autophagosomes) than lymphocytes and neutrophils. In each cell population, the mean number of vacuoles per cell (mean±SEM) is presented. **(B) and (E)**: Confocal microscopy (×1,000). LC3B fluorescence and mean LC3B fluorescence density is shown. CD34^+^ cells exhibit higher mean fluorescence density than lymphocytes and neutrophils. **(C) and (F)**: NanoPro1000^TM^ system. The LC3-I and LC3-II peaks and the ratio of the LC3-II peak area normalized to GAPDH is shown. CD34^+^ cells have higher levels of LC3-II than lymphocytes and neutrophils.

Because autophagy in CD34^+^ cells from AA patients appeared impaired, it was intriguing to study whether this phenomenon plays a role in the pathogenesis of AA. We therefore next investigated the autophagic characteristics of freshly sorted, healthy adult human bone marrow CD34^+^ cells that had not been cultured. Structural analysis via TEM showed that these cells exhibited more autophagic vacuoles (autophagosomes) in the cytoplasm than did lymphocytes and neutrophils **(Fig 4A)**. The mean number of autophagic vacuoles in CD34^+^ cells (7.60±3.13) was significantly higher than that in lymphocytes (2.50±1.43) and neutrophils (1.80±1.32) (*P =* 0.003) **(Fig 4D)**, suggesting a high autophagic activity in normal CD34^+^ cells. LC3B staining confirmed this result, as CD34^+^ cells exhibited a higher LC3B fluorescence density than did lymphocytes and neutrophils (*P<0*.*001*) **(Fig 4B and 4E)**. The same result was also supported by biochemical analysis, as we observed the conversion from cytosolic LC3-I to membrane-bound LC3-II **(Fig 4C and 4F)**.

### Rapid autophagic response in human bone marrow CD34^+^ cells to “adversity”

**Fig 5:**
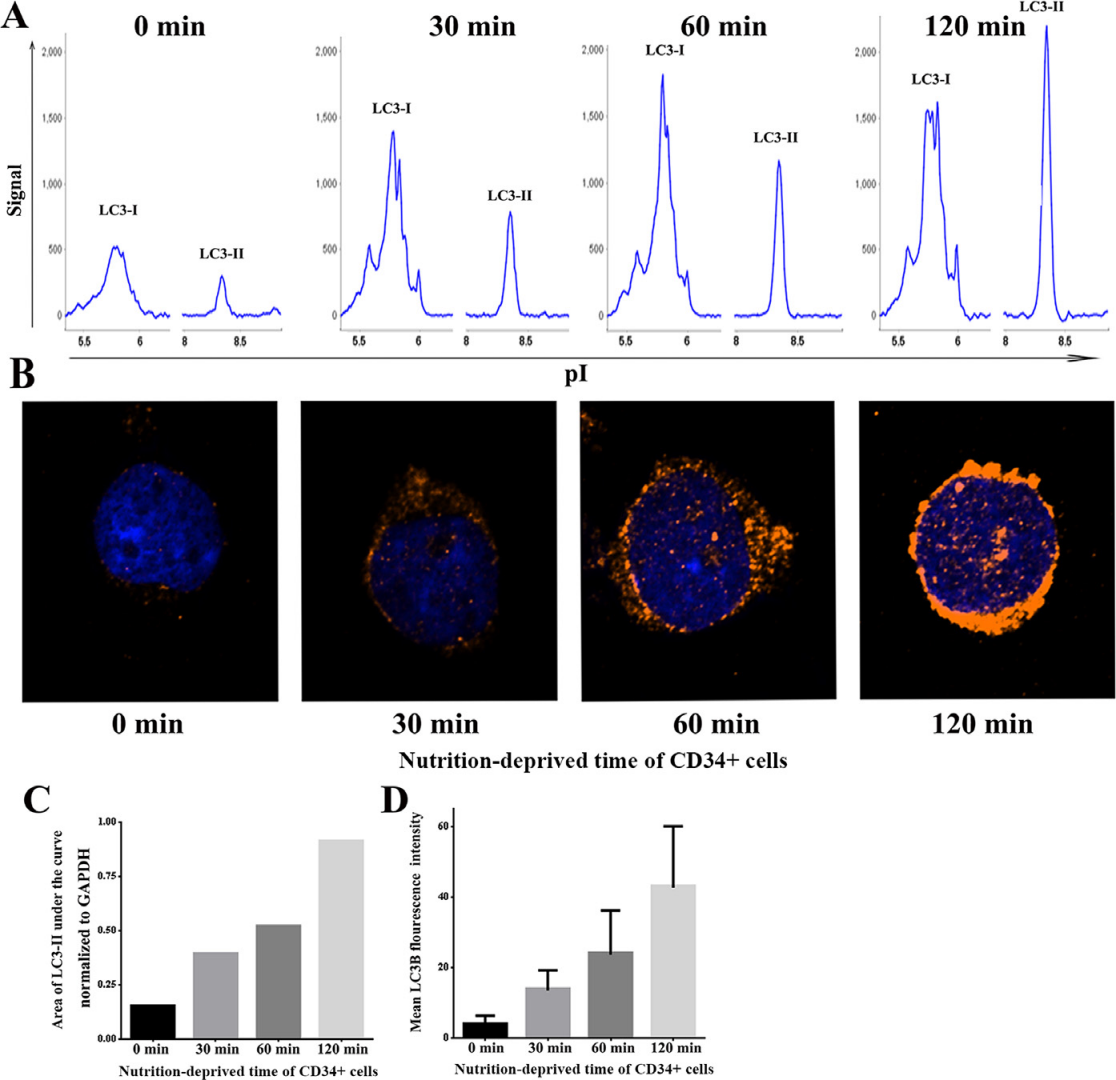
Levels of autophagy in healthy adult bone marrow CD34^+^ cells at different time-points of nutrient deprivation. Freshly sorted CD34^+^ cells were cultured for 12 h and placed in PBS for 0 min, 30 min, 60 min and 120 min after harvest. **(A) and (C)**: Analysis of LC3 protein using the NanoPro1000^TM^ system. The LC3-I and LC3-II peaks and the ratio of LC3-II peak area normalized to GAPDH at different time-points is shown. The LC3-II peak and peak area rapidly increased with the duration of nutrient deprivation. **(B) and (D)**: Analysis of LC3B using confocal microscopy. LC3B fluorescence and mean LC3B fluorescence density is presented. The LC3B fluorescence density in CD34^+^ cells was weak at 0 min and rapidly increased with the duration of nutrient deprivation.

Autophagy is known to be highly active in murine CD34^+^ cells **[33]**, and our study also found this process in freshly sorted CD34^+^ cells. The isolation of bone marrow CD34^+^ cells is time-consuming, and nutrition is deficient during the process. Because nutrient deprivation can induce cell autophagy, we next examined whether the high autophagic activity in CD34^+^ cells was simply a response to a nutrition-deprived state post-isolation. To do this, freshly isolated CD34^+^ cells from healthy human bone marrow were cultured for 12 h to correct potential effects of the isolation process on autophagy. Cells were then harvested and grouped, and different groups were incubated in PBS for 0 min, 30 min, 60 min and 120 min. Then, the levels of LC3 in the different groups were assessed using the NanoPro1000^TM^ system, an assay that could be initiated as soon as cells were harvested. As shown in **Fig 5A** and **5C**, the areas under the curve for LC3-II normalized to GAPDH at 0 min, 30 min, 60 min and 120 min were 0.15, 0.39, 0.52 and 0.91, respectively. The LC3-II in CD34^+^ cells was low at 0 min, suggesting that the basal autophagic activity of CD34^+^ cells was low. The level of LC3-II increased rapidly with increasing time in PBS (nutrient deprivation), suggesting that autophagy in CD34^+^ cells could be rapidly induced under unfavorable conditions. LC3B staining, which could also be initiated as soon as cells were harvested, confirmed this result. The mean LC3B fluorescence intensities at 0 min, 30 min, 60 min and 120 min were 3.70±2.79, 13.61±5.75, 23.87±12.52 and 42.84±17.41, respectively **(****Fig 5B** and **5D****)**. These results suggest that the basal autophagic activity of normal CD34^+^ cells is low and is induced dramatically under “adversity”.

### Autophagy is indispensable for proliferation and differentiation of human bone marrow CD34^+^ cells, and can protect them from apoptosis and death

**Fig 6:**
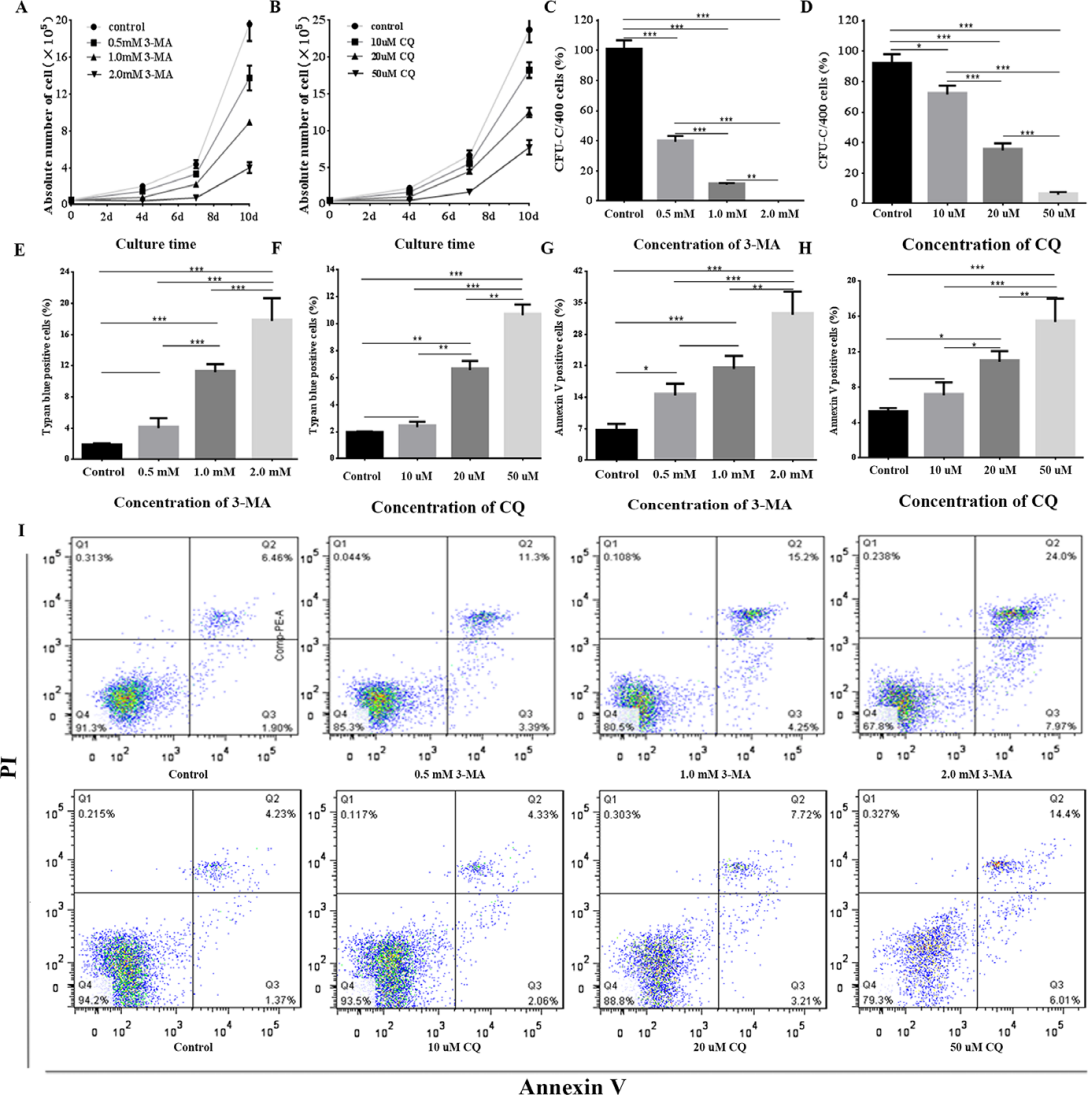
Autophagy effects on the survival of CD34^+^ cells. **(A) and (B):** CD34^+^ cells were treated with 3-MA or CQ, and the number of cells was counted at days 4, 7 and 10. 3-MA and CQ were able to inhibit proliferation of CD34^+^ cells in a concentration-dependent manner. The results shown are the mean±SEM of three independent experiments. **(C) and (D):** CFC assay. CD34^+^ cells were treated with 3-MA or CQ, and colonies were counted at day 10 and normalized to control conditions. 3-MA and CQ inhibited differentiation of CD34^+^ cells in a concentration-dependent manner. The results shown are the mean±SEM of three independent experiments. **(E) and (F):** Trypan blue assays revealing the percentage of dead cells. CD34^+^ cells were treated with 3-MA or CQ for 48 h prior to application of trypan blue. 3-MA and CQ induced the death of CD34^+^ cells in a concentration-dependent manner. The data represent the mean±SEM of three independent experiments. **(G-I)**: Apoptosis detection by AnnexinV-FITC/PI assays. CD34^+^ cells were treated with 3-MA or CQ for 24 h, followed by staining with Annexin-V/PI. 3-MA and CQ induced the apoptosis of CD34^+^ cells in a concentration-dependent manner. **G** and **H** represent the mean±SEM of three independent experiments, with the Q2 quadrant (Annexin V+/PI+) and Q3 quadrant (Annexin V+/PI-) in **I** representing the percentages of early and late apoptotic cells, respectively. * *P*<0.05, ** *P*<0.01, *** *P*<0.001.

Next, CD34^+^ cells were characterized by their capacity for proliferation and differentiation. To assess whether autophagy affects the survival of adult human bone marrow CD34^+^ cells, we used two autophagic inhibitors, 3-MA and CQ. The first is a phosphatidylinositol 3-phosphate kinase inhibitor and decreases autophagosomic LC3 (LC3-II), while CQ inhibits autophagy at a later stage by inhibiting fusion between autophagosomes and lysosomes **[45, 46]**. A total of 0.5×10^5^ sorted CD34^+^ cells were plated in culture and treated with 0 mM, 0.5 mM, 1.0 mM, or 2.0 mM 3-MA and 0 μM, 10 μM, 20 μM, or 50 μM CQ. At day 10, the cell numbers in the four 3-MA-treated groups were 19.6±3.2×10^5^, 13.8±2.3×10^5^, 8.9±0.5×10^5^ and 4.0±1.0×10^5^, respectively **(Fig 6A)**, while the four CQ-treated groups were 23.7±3.0×10^5^, 18.2±1.8×10^5^, 12.5±1.1×10^5^ and 7.7±1.7×10^5^, respectively **(Fig 6B)**. These results suggested that the autophagic inhibitors 3-MA and CQ can inhibit proliferation of CD34^+^ cells in a concentration-dependent manner and that autophagy can affect the proliferation of CD34^+^ cells.

Next we performed a CFC assay, in which 400 sorted CD34^+^ cells were seeded into methylcellulose culture medium containing 0 mM, 0.5 mM, 1.0 mM or 2.0 mM 3-MA and 0 μM, 10 μM, 20 μM, or 50 μM CQ. Colonies were counted at day 10, and the CFC numbers of the four 3-MA-treated groups were 52.7±3.5, 20.7±2.1, 5.7±0.6 and 0, respectively **(Fig 6C)**. In the four CQ-treated groups the CFC numbers were 48.0±3.6, 37.7±3.1, 18.3±2.5 and 3.0±1.0, respectively **(Fig 6D)**. These results indicate that 3-MA and CQ can also inhibit the proliferation of CD34^+^ cells in a concentration-dependent manner and that autophagy can also affect the proliferation of CD34^+^ cells.

We next attempted to study the effect of autophagy on the viability of CD34^+^ cells. Sorted CD34^+^ cells were incubated with 0 mM, 0.5 mM, 1.0 mM, or 2.0 mM 3-MA and 0 μM, 10 μM, 20 μM, or 50 μM CQ. Cell viability analysis using trypan-blue exclusion was carried out after 48 h. The percentage of dead cells in the four 3-MA-treated groups was 1.69±0.37%, 4.01±1.27%, 11.2±1.04% and 17.7±2.9%, respectively **(Fig 6E)**, and 1.87±0.15%, 2.35±0.40%, 6.57±0.68% and 10.6±0.8% in the four CQ-treated groups, respectively **(Fig 6F)**. These data suggest that 3-MA and CQ can induce the death of CD34^+^ cells in a concentration-dependent manner and that impaired autophagy can enhance this effect.

To further evaluate the effect of autophagy on the induction of apoptosis of CD34^+^ cells, the cells were treated with 0 mM/0.5 mM/1.0 mM/2.0 mM 3-MA and 0 μM, 10 μM, 20 μM, or 50 μM CQ. Annexin V-FITC/PI double staining and flow cytometry was then performed after 24 h. The proportion of apoptotic cells in the four 3-MA-treated groups was 6.39±1.72%, 14.4±2.6%, 20.3±2.9% and 32.4±5.2%, respectively, and in the four CQ-treated groups 5.10±0.53%, 7.08±1.47%, 10.9±1.2% and 15.4±2.6%, respectively. These results suggest that 3-MA and CQ could induce apoptosis of CD34^+^ cells in a concentration-dependent manner (**Fig 6G–6I**) and that impaired autophagy can sensitize CD34^+^ cells to apoptosis.

## Discussion

AA has been considered primarily as an immune-mediated bone marrow failure syndrome **[47]**. Profound quantitative and qualitative defects of hematopoietic stem and progenitor cells have been a consistent finding in AA **[5–11]**, although little is known about the responsible mechanisms. To systemically investigate autophagy in HPCs from AA patients, we compared levels of autophagy in bone marrow CD34^+^ HPCs between AA cases and age/sex-matched healthy controls. Our results identified significantly decreased autophagy in CD34^+^ cells from patients with *de novo* AA. In addition, CD34^+^ cells in SAA patients exhibited lower autophagy than did cells from NSAA patients, indicating that the autophagy in CD34^+^ cells from AA patients was dysfunctional. Based on the outcome of relatively large studies performed in the 1990s **[48]**, an ATG-based regimen combined with cyclosporine was most widely used to treat AA. With this treatment regime, the overall response rate (including CR and PR) to IST was 62–77%, but relapse occurred in 9.9% to 37% of the responded patients **[49–51]**. To examine the change in autophagy in CD34^+^ cells upon the amelioration of AA, we assessed autophagy in CD34^+^ cells in AA with CR, PR and NR, and found that autophagy in CD34^+^ cells improved with the amelioration of disease. However, compared to healthy controls, the mean autophagy in CD34^+^ cells from AA patients remained significantly lower even in AA patients who had achieved long-term CR. IST was unable to rescue autophagy in CD34^+^ cells, suggesting that dysfunctional autophagy may be intrinsic in CD34^+^ cells from AA patients.

Hematopoietic stem cells reside in a hypoxic niche, rely heavily on glycolysis and remain quiescent for extensive periods **[52, 53]**. Differentiated and committed progenitors rely on oxidative phosphorylation, and the transition from quiescence to proliferation/differentiation is accompanied by an increased metabolic rate, mitochondrial metabolism and ROS **[54, 55]**. Less is known about the mechanisms used by hematopoietic stem and progenitor cells to cope with cellular stress, however. Recent studies and our results all demonstrated that freshly sorted hematopoietic stem and progenitor cells had high autophagic activity **[33, 34]**. Additionally, autophagy-deficient hematopoietic stem and progenitor cells, created with a conditional knockout of *Atg7* or *FIP200*, accumulated mitochondrial mass and ROS **[33, 56]**. Furthermore, *ex vivo* cytokine withdrawal and *in vivo* calorie restriction could robustly induce autophagy driven by the transcription regulator *FOXO3* **[35],** and our results also showed that the autophagic response to nutrition deprivation in hematopoietic progenitor cells was very fast and robust. Together, these results suggest that autophagy is an important mechanism for the regulation of mitochondrial quantity and quality, as well as the balance of superoxide in hematopoietic stem and progenitor cells, and that a rapid and robust autophagy response is a protective mechanism used by hematopoietic stem and progenitor cells to cope with cellular stress.

Here, we found autophagy to be highly active in freshly sorted hematopoietic stem and progenitor cells. The isolation of CD34^+^ cells from mononuclear cells was time-consuming, and cells faced nutrient deprivation during isolation. Previous work has shown that nutrient deprivation induces autophagy **[57]**. To clarify whether hematopoietic stem and progenitor cells had high basal autophagy activity or had a rapid autophagic response to nutrient deprivation, we did not directly assess autophagy in freshly sorted CD34^+^ cells, but instead cultured cells for 12 h to reduce the effects of cell isolation. Furthermore, freshly harvested cells were incubated in PBS for 0 min, 30 min, 60 min and 120 min to create different nutrition deprivation states. Results of these experiments revealed low levels of basal autophagy in CD34^+^ cells, which is in contrast to all other related reports that stated the autophagic activity of CD34^+^ cells (from both human and mouse) was high **[33, 34]**. However, these studies showed only the autophagic characteristics of freshly sorted CD34^+^ cells and ignored the effects of the isolation process. The basal level of autophagy in CD34^+^ cells was low, but the autophagy response of these cells to stress was very rapid and could reach a very high level after only 2 h of nutrient deprivation.

Autophagy is important for the development of the blood system, as mice lacking the essential autophagy gene *Atg7* show loss of normal hematopoietic stem cell functions, and deregulated fetal and postnatal hematopoiesis **[33].** Autophagy is also essential for the life-long maintenance of hematopoietic stem cells **[35, 56]**. However, there have been very few studies investigating autophagy in adult human bone marrow HPCs. Our results here indicated that autophagy is indispensable for survival of human bone marrow CD34^+^ cells. We found that inhibition of autophagy in human CD34^+^ cells resulted in their reduced proliferation, and when autophagy was inhibited completely, CD34^+^ cells could not survive *in vitro*. Furthermore, impaired autophagy sensitized CD34^+^ cells to death and apoptosis.

Autophagy was necessary for survival of human bone marrow CD34^+^ cells and played a protective mechanism to cope with cellular stress. Both freshly sorted CD34^+^ cells from AA patients patients and cultured CD34^+^ cells from AA patients showed lower autophagic activity than did normal bone marrow CD34^+^ cells. Additionally, IST was insufficient to rescue this deficit, suggesting that dysfunctional autophagy in CD34^+^ cells from AA patients may be intrinsic. The bone marrow environment where CD34^+^ cells from AA patients reside is adverse, and the cells undergo attack from the immune system. Inhibitory cytokines (interferon-γ and tumor necrosis factor-α) in AA bone marrow have been shown to be significantly higher than in normal bone marrow and could induce apoptosis and death in CD34^+^ cells from AA patients**[58–60]**. CD34^+^ cells from AA patients require high levels of autophagy to protect them from unfavorable conditions, but in our work, these cells displayed the contradictory characteristic of impaired autophagy. This result suggests that dysfunctional autophagy may at least partly explain the quantitative and qualitative defects of CD34^+^ cells from AA patients and may play an important role in the pathogenesis of AA. In addition, AA HPCs are much more apoptotic than normal HPCs in the presence of inhibitory cytokines **[13, 14].** Autophagy primarily acts as a protective mechanism to prevent cell death; thus impaired autophagy may partly explain why AA HPCs die more readily under stress.

Here, we showed dysfunctional autophagy in CD34^+^ cells from AA patients, but the molecular mechanisms underlying this phenotype need further investigation. We demonstrated that the basal levels of autophagy in bone marrow CD34^+^ cells were low, the isolation of CD34^+^ could induce high autophagy activity, and freshly sorted CD34^+^ cells from AA patients had impaired autophagy. Additionally, the levels of LC3-I and LC3-II protein were low, suggesting that the production of LC3 was depressed under these conditions. Starvation and energy depletion modulate the autophagy signaling pathway though mammalian target of rapamycin (mTOR) and AMP-activated protein kinase (AMPK) **[61]**. Thus, insights into this signaling pathway may guide future research in this field.

## Conclusions

In summary, this study demonstrates that autophagy in CD34^+^ cells from AA patients is impaired, basal autophagy in bone marrow CD34^+^ cells is low, and the autophagic response of CD34^+^ cells to “adversity” is rapid. We also show that autophagy is necessary for the survival of CD34^+^ cells, and impaired autophagy in CD34^+^ cells may play a role in the pathogenesis of AA.
